# From Paper to Pixels: Evaluating the Impact of Digital Transformation on Sustainability in Nursing Education

**DOI:** 10.1155/jonm/6145329

**Published:** 2025-04-23

**Authors:** Pauletta Irwin, Amy Barnett, Kerryn Butler-Henderson, Mathew Ellis, Jeong-Ah Kim, Deborah Magee, Simon McDonald, Lisa Speedie, Shanna Fealy

**Affiliations:** ^1^School of Nursing, Paramedicine and Healthcare Sciences, Charles Sturt University, Locked Bag 5000, Port Macquarie 2444, New South Wales, Australia; ^2^School of Nursing, Paramedicine and Healthcare Sciences, Charles Sturt University, Locked Bag 558, Wagga Wagga 2678, New South Wales, Australia; ^3^School of Nursing, Paramedicine and Healthcare Sciences, Charles Sturt University, Panorama Avenue, Bathurst 2795, New South Wales, Australia; ^4^Spatial Data Analysis Network (SPAN), Charles Sturt University, Locked Bag 5000, Port Macquarie 2444, New South Wales, Australia; ^5^School of Nursing, Paramedicine and Healthcare Sciences, Charles Sturt University, P.O. Box 789, Albury 2640, New South Wales, Australia

## Abstract

**Background:** As the largest workforce in health, it is critically important that the principles of planetary health are incorporated into nursing education to have a significant influence on reducing the environmental impacts of health practices and address health inequities. Despite the adoption of sustainability principles by the higher education sector, the translation into nursing curriculum has been challenging. The increased integration of digital technologies in health contributes towards both sustainability in health and the United Nations Sustainable Development Goals (SDG). Electronic health records (EHRs) are well documented in reducing the reliance on paper-based records and improve the efficiency of health delivery, including accessibility to health where information sharing barriers are reduced. The incorporation of digital health training, and specifically the use of simulated EHRs, into nursing curriculum will increase the application of the principles of planetary health into graduate practice.

**Aims:** This study explored if the use of an EHR in a simulated learning environment (SLE) in a Bachelor of Nursing (BN) program can lead to tangible reduction in paper consumption.

**Methods:** A nonexperimental, pre- and postdesign was used to audit the paper consumption, specifically paper-based manuals, within the SLE pre- and postimplementation of the HealthiERSim®, an in house developed simulated EHR system. The study was held across multiple campuses with different BN cohorts.

**Results:** The study reported an overall reduction paper consumption by 12.59% post-implementation of HealthiERSim® and a reduction in pages per SLE manual, with a significant (*p* < 0.001) difference in paper consumption between the subjects.

**Conclusion:** The results of this study indicate an effective implementation of an EHR solution can decrease paperconsumption within an SLE, contributing to reducing the environmental footprint of an educational institution. Where adopted with curriculum content about sustainable practices, this will increase graduate awareness and practice of planetary health principles and the SDGs.

## 1. Introduction

The growing recognition of planetary health as a critical concept emphasises the complex relationship between human health and the sustainability of Earth's ecosystems. Planetary health is widely recognised as a comprehensive strategy that aims to attain the highest levels of global health, well-being, and equity while protecting the planet's natural systems [1]. This paradigm requires the melding of social, economic, and environmental policies that address health determinants, all within the constraints of environmental sustainability, to maintain humanity's long-term viability [2]. Environmental variables have a significant impact on health outcomes, emphasising the importance of this comprehensive approach.

Recent literature emphasises the importance of sustainability in healthcare, particularly as the sector continues to be a significant contributor to global greenhouse gas emissions [3]. The healthcare sector's role in environmental degradation has prompted calls for more sustainable practices, particularly in the education and training of future healthcare professionals. Nursing education plays a critical role in this transition as it prepares the next generation of healthcare workers to implement and advocate for environmentally responsible practices [4].

As universities and healthcare institutions increasingly acknowledge their responsibility for promoting sustainability, there is a growing emphasis on the integration of digital technologies within educational curricula. These technologies not only enhance learning outcomes but also contribute to the reduction of resource consumption, thereby aligning educational practices with broader sustainability goals [5]. This study explores the impact of incorporating digital tools into nursing simulation education, specifically assessing if a transition to electronic health records (EHRs) can reduce the environmental footprint within the simulated learning environment (SLE), while maintaining high standards of simulated educational practices.

## 2. Background

A central principal of planetary health is the nexus between the health of humans and the health of the planet, recognising that achieving health gains at the cost of eroding Earth's natural systems is unsustainable and detrimental to human civilization [6]. Planetary health is defined as the attainment of the highest standards of health, well-being, and equity worldwide through careful stewardship of the political economic and social systems, emphasising the deep interconnectedness of human and environmental health [7].

To achieve planetary health, integrated policies must be developed, implemented, and evaluated, addressing the social, economic, and environmental determinants of health [7]. This is intricately linked to the concept of sustainability, which highlights that while human health has improved, it has often been at the expense of the planet's health and the well-being of future generations. In response to this understanding, the United Nations Sustainable Development Goals (SDG) were established in 2015 and have 17 goals and 169 targets [8, 9]. Unsustainable patterns of resource consumption and technological development continue to exacerbate these challenges however, resulting in persistent inequitable and inefficient outcomes [7].

Within this framework, health systems serve as arenas where the sustainability of human health and the environment converge. Nurses, as frontline healthcare providers are uniquely positioned to lead efforts in reducing the environmental impacts of healthcare practices while simultaneously addressing health inequities [7, 10]. To fulfill this critical role, it is imperative that nursing education incorporates the principles of planetary health. By integrating environmental sustainability into nursing curricula, educational institutions can equip future nurses with the knowledge and skills necessary to promote both human and planetary health. This approach not only prepares nurses to engage in environmentally responsible healthcare practices but also positions them as advocates for policies that enhance the resilience of health systems and communities.

The tertiary and health sectors therefore hold significant responsibility in progressing the SDGs and planetary health. Through the creation and dissemination of knowledge, particularly in health and education, these sectors are uniquely positioned to support positive change and foster innovation, economic development, and societal well-being [10, 11].

### 2.1. Sustainability in Higher Education

Higher education institutions have a critical responsibility to promote sustainable development through both pedagogical efforts and research. This involves reducing carbon footprints and educating students on global sustainability issues, with initiatives like transforming campuses into “green campuses” demonstrating positive impacts on stakeholders' quality of life and offering strategic marketing advantages [12–14]. Despite the benefits, embedding sustainability into curricula remains challenging due to traditional discipline-specific focuses, necessitating a systemic and integrated approach to curriculum design [15, 16].

To effectively foster sustainability competencies in students, education must transition from a “bolt-on” to a “built-in” approach, where sustainability is incorporated across all functions and activities [17, 18]. Education for Sustainability (EfS) plays a crucial role in this transformation, equipping students as agents of change. Although slow progress and disciplinary barriers persist, the growing trend among educators to incorporate environmental awareness and social commitment into teaching, supported by sustainability champions, can help overcome these challenges and promote a more integrated approach to education [19, 20]. In this context, the inclusion of key sustainability competencies into higher education curricula is essential for enhancing students' capacities for personal growth, empathy, and societal transformation [19, 21].

### 2.2. Sustainability in Nursing Education

The educational preparation of nurses and midwives must include “mitigation of the health sector's impact on the environment, prepare for current and future health impacts of climate change, and advocate for policies and practices that promote ecosystem health and resilience” [10]. Intrinsic to educational preparation of nurses and other health professionals includes the recognition and integration of Indigenous knowledges, in particularly epistemologies [22]. This is consistent with the International Council of Nurses [23] Code of ethics for nurses which includes the requirement for nurses to contribute to the achievement of the SDGs.

One such critical technology is the adoption of EHRs. EHRs contribute to sustainability by reducing reliance on paper-based records and improving the efficiency of health care delivery [24]. Integrating EHRs into nursing education not only familiarises students with essential industry standard digital competency [25, 26] but also prepares them to contribute to more sustainable digital health practices in their professional careers [27]. This aligns with the broader SDGs, which emphasise the importance of technology innovations in fostering sustainable health and education practices [8].

A paradigm shift in nursing simulation is required, with greater efforts to incorporate more sustainable practices to reduce clinical waste within the SLE [28]. Incorporating digital health technologies such as EHRs into these areas demonstrates authentic practices and affirms a commitment to sustainability. By demonstrating sustainable practices through technology integration, nursing programs can prepare students for sustainable healthcare models.

Adopting value-informed nursing practices with a focus on environmental outcomes can position nurses as leaders of change in sustainable healthcare [29]. Nursing education plays a crucial role in this transformation by equipping future nurses with the knowledge and skills needed to implement sustainable practices. This includes addressing inequities in access to digital health technologies, particularly for marginalised communities, and educating students on designing and implementing accessible and equitable digital health solutions [30]. By doing so, nursing programs can empower nurses to become agents of change in their professional practice [31].

### 2.3. Objective of the Study

Building on the principles of planetary health, sustainability in higher education and specifically, nursing education, this study aims to explore if a transition to an EHR program within the SLE of a Bachelor of Nursing (BN) program can lead to tangible reductions in paper consumption.

### 2.4. Significance of the Study

The objective of the study aligns with the broader goal of promoting sustainable healthcare practices, which is crucial for both human and planetary health. By exploring the environmental impact of transitioning to EHRs, this study contributes to understanding how nursing education can incorporate sustainable practices, thereby preparing future nurses to be leaders in environmentally responsible care. The findings will offer valuable recommendations for future implementation and incorporation of digital technology tools across various educational programs.

## 3. Methods

This study employed a nonexperimental, pre- and postdesign to assess the effects of implementing the HealthiERSim® system on paper consumption within the SLE. An audit of paper based SLE manuals was conducted across all relevant BN curriculum subjects for years 2023 (pre-implementation) and 2024 (post-implementation). The primary objective was to understand if a transition to an EHR system can demonstrate real world reductions in paper consumption within the SLE and identify areas for continued quality improvement. The audit did not involve collecting student information or using human participants. All reported data, including the number of students, were anonymised, and subject names were de-identified, classifying this as a low-risk quality assurance activity. According to National Health and Medical Research Council (NHMRC) guidelines [32], such activities do not require ethics approval because they pose negligible risk and are conducted using de-identified data without direct human involvement.

### 3.1. Study Setting

The study was conducted across multiple campuses that deliver the BN program. Students enrolled in this program of study can choose between distance or on-campus study modes. Those enrolled in a distance mode, in general are required to attend an intensive week of skill acquisition within one of the five campus based SLE's. Those enrolled in an on-campus mode are required to participate in weekly skill acquisition sessions at their designated campus. Across all six key SLE subjects within the BN program, all students are required to use paper-based SLE manuals. These manuals contain essential learning materials, including patient simulation scenarios, reflective practice prompts, and various charts for simulated patient documentation.

### 3.2. Intervention

Prior to the commencement of SLE classes in early 2024, an EHRs system called HealthiERSim® was introduced to enhance industry relevant digital literacy of nursing students within the SLE. HealthiERSim® is an in-house developed EHRs simulation system designed to mimic real-world digital documentation used in clinical practice. It allows students to document patient assessments, nursing interventions, and care plans within the SLE. HealthiERSim® was introduced to replace traditional paper-based records, providing a more authentic learning experience while promoting sustainability. The HealthiERSim® software system was informed by research conducted by the designers and developed at a regional Australian university with additional goals to enhance simulated teaching and learning practices and reduce paper consumption aligned with UN SDGs. Mobile workstations equipped with a laptop with the HealthiERSim® software were made available for student use within the SLE, allowing nursing educators to adopt the use of EHR within their SLE teaching practices so students are exposed to using EHR systems prior to engaging in industry workplace learning. The adoption of HealthiERSim® varied across SLE subjects.

### 3.3. Data Collection

To test the effectiveness of adopting HealthiERSim® on paper consumption, an audit of SLE manuals was undertaken for both 2023 (preintervention) and 2024 (postintervention), to quantify and compare paper consumption. The paper consumption audit was conducted by researcher AB, with the number of pages per SLE manual (for both distance and on campus students) for each of the six relevant SLE subjects collected for both 2023 and 2024. The total number of enrolled students for each subject was also collected to assist with making comparisons as per Supporting File 1. Data were compiled in a spreadsheet for analysis, with descriptive and inferential statistics applied to assess changes in paper consumption.

### 3.4. Data Analysis

Count data derived from the audit was summarised and presented descriptively. To statistically evaluate the differences in paper usage across subjects due to the implementation of HealthiERSim® between 2023 and 2024, a Pearson's Chi-squared test was performed on the total paper consumption by subjects. This test assessed whether there was a significant dependence on the grouping of the data (i.e., whether the paper usage in 2023 and 2024 differed significantly across subjects). An additional posthoc analysis was conducted to identify which specific subjects contributed to reductions and to identify where further EHR adoption within subjects may be required. Descriptive statistics were calculated using Microsoft Excel version 16.87 with inferential statistics conducted using R programming language version 4.2.3 and with the assistance of a statistician.

## 4. Results

The summarised data obtained from the audit is presented in Table 1. Overall reductions in pages per SLE manual were evident across all distance manuals. For on-campus manuals reductions in pages were seen for subjects NS01, NS03, NS05, and NS06 with an increase in pages seen for NS04 suggesting varied EHR adoption. Overall paper consumption decreased by 12.59% from 2023-2024.

Table 2 presents the total paper consumption (lab manual pages) by enrolled student numbers across subjects for 2023, and 2024. The results of a Pearson's Chi-squared test applied to this data show a strong dependence on how the data is grouped, indicating significant differences in paper consumption between the subjects. The result of the post hoc analysis presented in Table 3 helps to identify the subjects where statistically significant reductions or increases in paper consumption were observed between 2023 and 2024. Residuals reflect the difference between the observed and expected counts. A positive residual in 2023 paired with a negative residual in 2024 suggests that paper consumption was higher in 2023 but decreased in 2024, suggestive of successful EHR adoption leading to reductions in paper consumption. A negative residual in 2023 followed by a positive residual in 2024 indicates an increase in paper consumption and identifies subjects for quality improvement. Statistically significant reductions in paper consumption were observed for NS03 *p* < 0.001) and NS05 (*p* < 0.001). Significant increases in paper consumption were evidenced for subjects NS01, NS04, and NS06 (*p* < 0.001) with no differences in paper consumption observed for NS02.

## 5. Discussion

This study aimed to evaluate the adoption of the HealthiERSim® system within six SLE subjects in one Australian BN program. The results indicate a complex pattern of paper consumption across subjects with statistically significant reductions and increases in paper consumption observed. To the authors knowledge, this is the first research that has attempted to measure the use of paper post initial implementation of a simulated EHR system as a measure of sustainability.

The most substantial reductions in paper consumption were observed for NS03 and NS05 whereby the adoption of HealthiERSim® led to a significant decrease in SLE manual pages from 2023 to 2024 by *n = *14,448 and *n = *66,496, respectively. When tested under real-world conditions the reduction in paper consumption for NS05 dropped from 107,500 pages (*n = *500 students) in 2023 to 41,004 pages (*n = *612 students) in 2024, even with an increase in student enrolments between years (*p* < 0.001). Similarly, NS03 saw a drop in paper consumption from *n = *87, 608 in 2023 to *n = *73,160 in 2024, observing significant reductions in paper consumption, again when student enrolments increased over time (*p* < 0.001). These findings suggest that when effectively adopted, digital resources can decrease paper-consumption within the SLE, contributing to sustainable education practices. The reduction in paper usage also aligns with the broader objective of minimising the environmental footprint of educational institutions, which is a crucial aspect of planetary health and sustainability [5].

The results have also identified areas for improvement. Unexpected increases in paper consumption were evident for subjects NS01 and NS06 where paper consumption increased between timepoints even with a decrease in SLE manual pages (*p* < 0.001). It is possible that HealthiERSim® was only partially adopted within these subjects and with an increase in student enrolments from 2023 to 2024 saw significant increases in paper consumption as more students required access to printed materials. These findings highlight the challenges associated with transitioning to digital systems in educational settings with the need for approaches that ensures all aspects of the curriculum are aligned with the digital tools being implemented. Further investigation into the reasons behind the increases in lab manual pages for such as NS04 and the relatively unchanged NS02 is warranted, with consideration of and targeted interventions to address the challenges associated with the adoption of the HealthiERSim® system.

The mixed results of this study reflect the complexities of integrating sustainability practices into educational programs. While the adoption of HealthiERSim® has proven beneficial in reducing paper in certain subjects, the overall effectiveness of such initiatives depends on the level of adoption and integration and the specific contexts of each subject. For successful sustainability outcomes, it is essential that digital tools are fully embraced across the curriculum, with sufficient support and training provided to both educators and students. A systematic approach is recommended to successfully integrate sustainable approaches in education [15, 16]. Further research is needed to explore the barriers and enablers to successful digital integration in subjects where paper usage increased. Qualitative studies involving educators and students could provide insights into their experiences with the HealthiERSim® system and identify areas for improvement. Additionally, future studies could evaluate the long-term impact of digital tools on educational outcomes, as well as their sustainability and economic benefits.

### 5.1. Limitations

This study provides valuable insights into the transition from paper-based resources to an EHR system within a BN program however, several methodological limitations should be noted. The time period of one year may not fully capture long-term effects and behavioural changes among students and educators during the initial adoption and transition phase, requiring a longer time period and support. The small data sample and reliance on manual data collection by one researcher may have introduced errors and may not reflect the total paper consumption accurately. The study is also limited to a single regional Australian university, which means that findings cannot be generalised outside of this context. Institutional differences, variations in curriculum design, and digital resource availability may influence the effectiveness of transitioning to EHRs. Future collaborations with other universities would provide a broader perspective on the impact of digital health records on sustainability in nursing education. Lastly, the lack of qualitative data means that the experiences and attitudes of students and educators were not captured, limiting the understanding of potential barriers to the adoption and transition to an EHR system. Future research should incorporate interviews or surveys to explore these concepts from a broader perspective.

## 6. Conclusion

The transition to the HealthiERSim® system within the BN program has demonstrated potential in reducing paper usage, particularly in subjects where full incorporation was achieved. However, the increase in paper usage in certain subjects underscores the need for a more cohesive and comprehensive approach to digital integration. By addressing these challenges, the BN program can continue to advance its sustainability goals and contribute to the broader discourse on sustainable practices in health professions education.

## Figures and Tables

**Table 1:** Comparison of paper consumption generated pre- and postimplementation of HealthiERSim®.

Subject code	Pages per lab manual by study mode		Student enrolments by study mode		Paper consumption by study mode		Total paper consumption
	Distance	On-campus	Distance	On-campus	Distance	On-campus	Overall
*Paper consumption 2023*							
NS01	70	70	314	320	21,980	22,400	44,380
NS02	93	72	271	315	25,203	22,680	47,883
NS03	94	94	640	292	60,160	27,448	87,608
NS04	147	140	314	221	46,158	30,940	77,098
NS05	215	215	265	235	56,975	50,525	107,500
NS05	156	156	232	199	36,192	31,044	67,236
Total	775	747	2036	1582	246,668	185,037	431,705

*Paper consumption 2024*							
NS01	65	65	429	346	27,885	22,490	50,375
NS02	72	72	280	305	20,160	21,960	42,120
NS03	59	59	828	412	48,852	24,308	73,160
NS04	145	145	496	272	71,920	39,440	111,360
NS05	67	67	416	196	27,872	13,132	41,004
NS05	123	123	313	173	38,499	21,279	59,778
Total	531	531	2762	1704	235,188	142,609	377,797

**Table 2:** Comparison of total pages consumed by student subject enrolments, pre-, and postimplementation of HealthiERSim®.

Subjects	Overall paper consumption 2023	Overall paper consumption 2024	Difference in paper consumption 2023–2024	Pearson's Chi-squared test (*p* < 0.05)
NS01	44,380	50,375	−5995	***p* < 0.001**
NS02	47,883	42,120	5763	
NS03	87,608	73,160	14,448	
NS04	77,098	111,360	−34262	
NS05	107,500	41,004	66,496	
NS05	67,236	59,778	7458	
NS06	**431,705**	**377,797**	**53,908**	

*Note:* The bold numbers are the bottom are the totals for each column.

**Table 3:** Post hoc analysis.

Subject	Residual 2023	*p* value	Residual 2024	*p* value
NS01	−42.64	< 0.001	42.64	< 0.001
NS02	−0.82	1.00	0.82	1.00
NS03	10.45	< 0.001	−10.45	< 0.001
NS04	−123.39	< 0.001	123.39	< 0.001
NS05	162.92	< 0.001	−162.92	< 0.001
NS05	−3.06	0.03	3.06	0.03

## Data Availability

The data that support the findings of this study are available from the corresponding author upon reasonable request.

## Supporting Information

This table shows the data pre- and post-HealthiERSim® project. The change in the number of pages in the Simulated Learning Environment Manual (SLE) manual for each subject is shown for 2023 and 2024. The subject code is the left column followed by pages per lab manual for distance and on-campus subjects. pages in each SLE manual. The total number of enrolled students for each subject was also collected. This data informs the results of paper consumption per cohort per session for each subject in Table 1 of the manuscript.
