# Supplementary material for: From Paper to Pixels: Evaluating the Impact of Digital Transformation on Sustainability in Nursing Education
Source: J Nurs Manag. 2025 Apr 23;2025:6145329. doi: 10.1155/jonm/6145329 (PMC12043383; doi:10.1155/jonm/6145329)
Supplement: Supporting Information — Additional supporting information can be found online in the Supporting Information section. [file 6145329.f1.docx]

**Supplementary file 1: Raw Audit Results**

| **2023**  **(pre - HealthiERSim)** | | | | |
| --- | --- | --- | --- | --- |
| **Subject code** | **Pages per lab manual**  **(Distance)**  ***n =*** | **Pages per lab manual**  **(On-campus)**  ***n =*** | **Student enrolments**  **(Distance)**  ***n =*** | **Student enrolments**  **(On-campus)**  ***n =*** |
| NRS163 | 70 | 70 | 314 | 320 |
| NRS174 | 93 | 72 | 271 | 315 |
| NRS277 | 94 | 94 | 640 | 292 |
| NRS283 | 147 | 140 | 314 | 221 |
| NRS387 | 215 | 215 | 265 | 235 |
| NRS399 | 156 | 156 | 232 | 199 |
| **2024**  **(post- HealthiERSim)** | | | | |
| NRS163 | 65 | 65 | 429 | 346 |
| NRS174 | 72 | 72 | 280 | 305 |
| NRS277 | 59 | 59 | 828 | 412 |
| NRS283 | 145 | 145 | 496 | 272 |
| NRS387 | 67 | 67 | 416 | 196 |
| NRS399 | 123 | 123 | 313 | 173 |

This table shows the data pre and post HealthiERSim project. The change in the number of pages in the Simulated Learning Environment Manual (SLE) manual for each subject is shown for 2023 and 2024. The subject code is the left column followed by pages per lab manual for Distance and On-campus subjects. pages in each SLE manual. The total number of enrolled students for each subject was also collected. This data informs the results of paper consumption per cohort per session for each subject in Table 1 of the manuscript
